# Attention-Based PSO-LSTM for Emotion Estimation Using EEG

**DOI:** 10.3390/s24248174

**Published:** 2024-12-21

**Authors:** Hayato Oka, Keiko Ono, Adamidis Panagiotis

**Affiliations:** 1 Master’s Program in Information and Computer Science, Doshisha University, Kyoto 610-0394, Japan; 2 Department of Intelligent Information Engineering and Sciences, Doshisha University, Kyoto 610-0394, Japan; kono@mail.doshisha.ac.jp; 3 Department of Information and Electronic Engineering, International Hellenic University, 57001 Thessaloniki, Greece; adamidis@ihu.gr

**Keywords:** emotion estimation, EEG, LSTM, PSO, attention mechanism, DEAP, SEED, four-class classification, three-class classification

## Abstract

Recent advances in emotion recognition through Artificial Intelligence (AI) have demonstrated potential applications in various fields (e.g., healthcare, advertising, and driving technology), with electroencephalogram (EEG)-based approaches demonstrating superior accuracy compared to facial or vocal methods due to their resistance to intentional manipulation. This study presents a novel approach to enhance EEG-based emotion estimation accuracy by emphasizing temporal features and efficient parameter space exploration. We propose a model combining Long Short-Term Memory (LSTM) with an attention mechanism to highlight temporal features in EEG data while optimizing LSTM parameters through Particle Swarm Optimization (PSO). The attention mechanism assigned weights to LSTM hidden states, and PSO dynamically optimizes the vital parameters, including units, batch size, and dropout rate. Using the DEAP and SEED datasets, which serve as benchmark datasets for emotion estimation research using EEG, we evaluate the model’s performance. For the DEAP dataset, we conduct a four-class classification of combinations of high and low valence and arousal states. We perform a three-class classification of negative, neutral, and positive emotions for the SEED dataset. The proposed model achieves an accuracy of 0.9409 on the DEAP dataset, surpassing the previous state-of-the-art accuracy of 0.9100 reported by Lin et al. The model attains an accuracy of 0.9732 on the SEED dataset, recording one of the highest accuracies among the related research. These results demonstrate that integrating the attention mechanism with PSO significantly improves the accuracy of EEG-based emotion estimation, contributing to the advancement of emotion recognition technology.

## 1. Introduction

In recent years, there has been growing interest in developing Artificial Intelligence (AI) systems capable of accurately recognizing human emotions. This technology holds immense potential for applications in advertising, healthcare, and autonomous driving, among others [1]. For example, in media effect studies used in advertising and promotion, human emotional values can serve as effective evaluation criteria [2,3]. Additionally, in the medical field, they can be utilized to support the diagnosis of diseases such as depression, dementia, and post-traumatic stress disorder (PTSD), as well as to assess stress and conditions in daily life [4,5]. In the field of autonomous driving technology, recognizing the emotions of the driver and combining them with information such as traffic and weather can enable the provision of optimal services [6,7].

Various approaches have been explored for emotion recognition, utilizing human facial expressions, voices, gestures, and texts [8]. Among these, emotion estimation using biosignals is particularly promising [9,10]. Unlike facial expressions, gestures, and voices, biosignals are difficult to intentionally disguise, leading to potentially more accurate emotion estimation [11,12]. In particular, electroencephalogram (EEG) has become a popular non-invasive evaluation technique that is safe and easy to administer, leading to advancements in research exploring its relationship with emotions.

Various models such as Long Short-Term Memory (LSTM), Convolutional Neural Network (CNN), and K-Nearest Neighbors (KNN) have been investigated for emotion estimation using EEG data [13,14,15,16]. However, these models often use fixed parameter settings, hindering their performance due to the insufficient optimization of parameters based on data characteristics. This highlights the need for methods that dynamically optimize model parameters. To address these limitations, we introduce Particle Swarm Optimization (PSO) to optimize the parameters of the LSTM model (number of units, batch size, dropout rate). PSO is a metaheuristic algorithm based on swarm intelligence, capable of efficiently finding optimal solutions in the entire search space [17]. This allows for the more accurate capture of complex patterns in EEG data [18].

Furthermore, to focus on the temporal and spatial features of EEG data, we incorporate an attention mechanism. The attention mechanism assigns weights to important parts of the input data, enabling the model to focus on significant information. This is expected to enhance the accuracy of emotion estimation by emphasizing the important features related to emotions in EEG data. By combining these techniques, we aim to improve the accuracy of emotion estimation based on EEG data.

This study focuses on the challenging task of four-class emotion classification, aiming to differentiate between high and low valence combined with high and low arousal states, with the goal of constructing a personalized model for each individual using Particle Swarm Optimization (PSO). Accurately classifying these emotional dimensions is crucial for effectively understanding and responding to human emotional states [19]. To achieve this, we utilize two widely used datasets for human affective state analysis: the DEAP and SEED datasets. The DEAP dataset contains EEG and peripheral physiological signals recorded from 32 participants as they watched 40 one-minute-long video clips designed to elicit different emotional responses [20]. The participants rated each video based on valence, arousal, liking, and dominance, allowing for a comprehensive analysis of emotional states. The SEED dataset comprises EEG data recorded from 15 subjects while watching 15 video clips, each approximately 4 min long [21,22]. The experiments were conducted three times for each subject, and each video was labeled as negative, neutral, or positive. Both datasets include preprocessed EEG signals, which are downsampled, filtered, and segmented into epochs, making them suitable for various emotion recognition tasks.

The remainder of this paper is organized as follows: Section 2 reviews the previous research on emotion estimation using EEG. Section 3 outlines the foundational technologies of this study, including LSTM, PSO, and the attention mechanism. Section 4 covers the preprocessing methods for EEG data and the attention-based PSO-LSTM model. Section 5 presents the experimental content, results, and discussion. Section 6 discusses the limitations of our approach and potential directions for future research. Finally, Section 7 provides a conclusion.

## 2. Related Research

EEG-based emotion recognition has seen significant advancements in recent years, with researchers exploring various datasets, methodologies, and learning techniques to improve emotion classification accuracy. This section discusses the key trends, challenges, and contributions from prior work, focusing on how they align with and differ from the current study.

Most studies utilized well-known datasets like DEAP and SEED, ensuring comparability of the results. However, the performance of these datasets can vary significantly based on the methodology and preprocessing techniques employed. The DEAP dataset, which includes EEG signals from 32 participants watching one-minute emotional video clips, is widely regarded for its balanced emotional stimuli and comprehensive annotations. Studies such as those of Marjit et al. (2021), Ajith et al. (2021), Nandi et al. (2021), and Bazargani et al. (2023) evaluated their methods for four-class emotion classification using the DEAP dataset [23,24,25,26]. Lin et al. (2023) and Deng et al. (2021) conducted evaluations using both the DEAP and SEED datasets [27,28], showing the effectiveness of validation across multiple datasets. The SEED dataset provides EEG recordings of subjects watching emotional film clips categorized into positive, neutral, and negative states. While both datasets aim to capture emotional states, they offer different characteristics: DEAP features shorter, varied clips with four-class classification, while SEED emphasizes longer-term responses with three-class classification.

We utilize DEAP and SEED datasets in this work to thoroughly validate our proposed PSO-LSTM approach. This dual-dataset evaluation strategy allows us to demonstrate the robustness and generalizability of our method across different experimental conditions, emotional categorizations, and temporal scales. By testing both datasets, we can provide a more comprehensive assessment of our model’s performance compared to existing benchmarks.

Most studies have focused on classifying emotions using the valence–arousal model, typically employing either binary (valence–arousal) or four-class emotion classification approaches.

A clear trend has emerged toward employing deep learning models to capture the spatiotemporal dynamics of EEG signals. Studies used handcrafted features such as Power Spectral Density (PSD) as seen in the work of Marjit et al. (2021). They utilized Power Spectral Density (PSD) features with a Genetic Algorithm-optimized Multi-Layer Perceptron (MLP), achieving high accuracies of 91% for binary classification and 83.52% for four-class classification. More recent efforts, like those of Deng et al. (2021) and Lin et al. (2023), leveraged advanced neural networks (e.g., CNNs and GNNs) to automatically learn the spatial and temporal features from raw EEG data. Their 3D Convolutional Neural Network approach achieved binary classification accuracies of 92.49% for valence and 91.94% for arousal, with a four-class classification accuracy of 74.23%. Lin et al. (2023) employed graph neural networks to extract intra- and inter-channel EEG features, demonstrating the importance of functional connectivity. Their approach achieved impressive four-class classification accuracies of 90.74% on the DEAP-Twente dataset and 91% on the DEAP-Geneva dataset. Bazargani et al. (2023) presented a lightweight deep learning model for emotion recognition, emphasizing binary classification. Their subject-dependent method achieved exceptionally high accuracies of 99.10% for valence and 99.20% for arousal, while the subject-independent method yielded accuracies around 90.76% for valence and 90.94% for arousal. Kan et al. (2023) proposed a self-supervised group meiosis contrastive learning framework that reduces reliance on labeled data [29]. Their binary classification results showed accuracies of 94.72% for valence and 95.68% for arousal. Ajith et al. (2021) emphasized the significance of Convolutional Neural Networks (CNNs) for temporal feature extraction, focusing on classifying emotions into valence and arousal classes.

Multiple works highlight the importance of accounting for inter-subject variability. For example, Bazargani et al. (2023) demonstrated significantly higher accuracy in subject-dependent setups, a finding echoed by Lin et al. (2023). Such variability emphasizes the need for approaches like PSO, which dynamically optimize model parameters for individual subjects.

Kan et al. (2023) addressed the challenge of limited labeled data using contrastive learning, showcasing the potential for semi-supervised methods to reduce dependency on extensive labeling, a recurring limitation in the field.

Some studies extended EEG-based emotion recognition to real-time contexts, emphasizing practical applications such as e-learning. For instance, Nandi et al. (2021) proposed a real-time emotion classification system designed for educational environments, which processed EEG data streams to recognize emotional states in dynamic settings.

A recurring issue in EEG-based emotion recognition lies in the lack of standardization in testing and validation. Studies vary in their use of subject-dependent or subject-independent setups and cross-validation strategies. For instance, while Bazargani et al. (2023) reported results under both setups, Marjit et al. (2021) primarily focused on subject-dependent approaches. Such differences complicate the direct comparisons between methods. This study mitigates these challenges by maintaining a consistent evaluation protocol on both DEAP and SEED datasets, aligning with the most common practices in the field.

Unlike prior efforts that often relied on fixed model architectures or limited optimization strategies, this study employs advanced optimization techniques to dynamically adapt to individual subject variations. By maintaining a consistent evaluation protocol and focusing on maximizing four-class classification accuracy, the research aims to address existing methodological challenges and contribute to the evolving landscape of EEG-based emotion recognition.

## 3. Related Technology

### 3.1. Long Short Term Memory

Long Short-Term Memory (LSTM) is a kind of Recurrent Neural Network (RNN) model that is capable of processing time series data and was proposed by Hochreiter and Schmidhuber in 1997 [17]. Compared to conventional RNNs, LSTM can store time-series data for extended periods. Due to its specialization in handling time-series data, LSTM has been widely used in the field of emotion estimation using EEG data [30,31]. In RNNs, the gradient of the objective function is used to update the parameters when learning them by backpropagation, but there is a problem, where the gradient disappears when the number of iterations exceeds a specific number. On the other hand, LSTM mitigates this issue by using a cell state that is updated through linear summation, which helps prevent excessive fluctuation of the gradient. While LSTMs still utilize sigmoid and tanh functions within their gates, the linear update of the cell state is key in preventing gradient loss [32,33].

**Figure 1:**
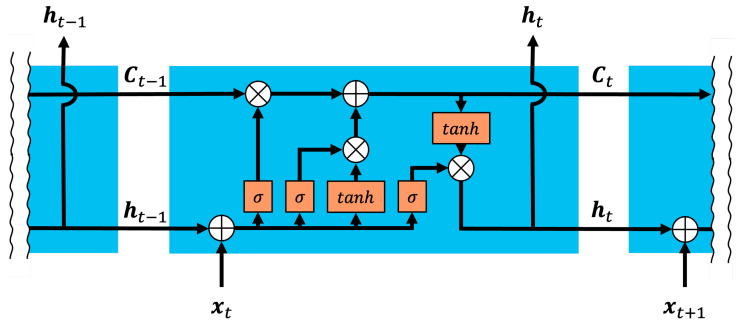
Internal LSTM structure illustrating the flow of hidden and cell states.

Figure 1 illustrates the internal structure of an LSTM. In this context, $h_{t}$, $c_{t}$, and $x_{t}$ represent the hidden state at time *t*, the cell state, and the input vector at time *t*, respectively. Additionally, σ and tanh denote the sigmoid and hyperbolic tangent functions, respectively. LSTM has three gates: a forget gate, an input gate, and an output gate, which enable long-term memory storage. The hidden state $h_{t}$ of LSTM is calculated as follows:

(1) $$\begin{array}{ccc} f_{t} & = & \sigma (W_{if}x_{t}+W_{hf}h_{t-1}+b_{f}) \end{array}$$

(2) $$\begin{array}{ccc} i_{t} & = & \sigma (W_{ii}x_{t}+W_{hi}h_{t-1}+b_{i}) \end{array}$$

(3) $$\begin{array}{ccc} o_{t} & = & \sigma (W_{io}x_{t}+W_{ho}h_{t-1}+b_{o}) \end{array}$$

(4) $$\begin{array}{ccc} \tilde{c_{t}} & = & \tanh(W_{if}x_{t}+W_{hf}h_{t-1}+b_{f}) \end{array}$$

(5) $$\begin{array}{ccc} c_{t} & = & f_{t}\circ c_{t-1}+i_{t}\circ \tilde{c_{t}} \end{array}$$

(6) $$\begin{array}{ccc} h_{t} & = & o_{t}\circ \tanh\left(c_{t}\right) \end{array}$$

where $x_{t}$ and $c_{t}$ represent the input vector and cell state, respectively, while W and b are the weight matrix and bias. In Equation (1), the forget gate vector $f_{t}$ is a weight vector that expresses how much of the previous cell state should be forgotten. In Equation (2), the input gate vector $i_{t}$ is a weight vector that expresses how much information should be added to the cell state $c_{t}$. In Equation (4), the candidate cell state $\tilde{c_{t}}$ is computed using the tanh function, which introduces nonlinearity into the cell state. These two gates together allow the cell state $c_{t}$ to be updated as a linear combination of the previous cell state and the candidate cell state $\tilde{c_{t}}$ as shown in Equation (5). In Equation (3), the output gate vector $o_{t}$ is the weight vector that determines how much information from the cell state $c_{t}$ should be transferred to the hidden state $h_{t}$. Finally, in Equation (6), the cell state $c_{t}$ is passed through a tanh function and modulated by the output gate to produce the hidden state $h_{t}$.

### 3.2. Particle Swarm Optimization

Particle Swarm Optimization (PSO) is a heuristic optimization algorithm inspired by the social behavior of organisms, particularly bird flocking or fish schooling [34,35]. Introduced by Kennedy and Eberhart in 1995, PSO aims to iteratively improve a candidate solution’s fitness by mimicking the social interactions among particles in a multidimensional search space [36]. In PSO, a population of potential solutions, termed “particles”, moves through the search space, guided by their own best-known position $p_{i}\left(t\right)$ and the global best-known position g(t) of the entire swarm. Each particle adjusts its position $x_{i}\left(t+1\right)$ and velocity $v_{i}\left(t+1\right)$ based on these two factors, aiming to converge towards optimal solutions. Position $x_{i}\left(t+1\right)$ and velocity $v_{i}\left(t+1\right)$ are calculated as follows:

(7) $$\begin{array}{cc} v_{i}\left(t+1\right) & =wv_{i}\left(t\right)+c_{1}r_{1}\left(p_{i}\left(t\right)-x_{i}\left(t\right)\right)\\ & \;+c_{2}r_{2}\left(g\left(t\right)-x_{i}\left(t\right)\right) \end{array}$$

(8) $$\begin{array}{cc} x_{i}\left(t+1\right) & =x_{i}\left(t\right)+v_{i}\left(t+1\right) \end{array}$$

where $r_{1}$ and $r_{2}$ are uniformly distributed random numbers in the range [0, 1], generated at each iteration. In Equation (7), the velocity $v_{i}\left(t+1\right)$ is calculated by a linear combination of the particle’s momentum, the cognitive component (personal best-known position $p_{i}\left(t\right)$), and the social component (global best-known position g(t)). PSO has shown potential in finding optimal solutions for loss functions with multimodal features by helping to avoid local solutions, although it does not guarantee global optimality.

### 3.3. Attention Mechanism

The attention mechanism is one of the techniques used in the field of machine learning and natural language processing, particularly useful when processing sequential data such as text or time series. The attention mechanism improves model performance by allowing the model to focus on essential parts of the input data, effectively mimicking how humans pay selective attention to specific pieces of information when processing a large amount of data [37]. In this paper, we introduce an attention mechanism to calculate the weight vector for each hidden state of an LSTM. This allows the model to determine which sequence parts are most relevant at each step of the computation [38]. The attention mechanism is calculated as follows:

(9) $$\begin{array}{ccc} \mathrm{score}(h_{t},h_{n}) & = & v_{a}^{⊤}\tanh\left(W_{a}\left[h_{t};h_{n}\right]\right) \end{array}$$

(10) $$\begin{array}{ccc} a_{t} & = & \mathrm{softmax}(\mathrm{score}\left(h_{t},h_{n}\right)) \end{array}$$

(11) $$\begin{array}{ccc} c & = & \sum_{t=1}^{n}a_{t}*h_{t} \end{array}$$

In Equation (9), a score function is defined to compute the relevance (or association) between the hidden state $h_{t}$ at each time *t* and the hidden state $h_{n}$ at the final time step *n*. In this score function, the hidden states $h_{t}$ and $h_{n}$ are combined and linearly transformed using the matrix $W_{a}$. Then, a bias term $v_{a}$ is added to it, and the tanh function is applied to obtain the final score. In Equation (10), the attention weight $a_{t}$ at each time step *t* is calculated using the score function, which computes the association between the hidden state $h_{t}$ and the final hidden state $h_{n}$ at the last time *n*. In Equation (11), the context vector c is created by taking a weighted sum of the hidden states $h_{t}$. The weights $a_{t}$ (from Equation (10)) determine how much attention the model gives to each time step when forming the context vector. This context vector c is then used by the model to make predictions.

## 4. Methodology

### 4.1. DEAP Dataset

The Database for Emotion Analysis using Physiological Signals (DEAP) is a dataset for emotion analysis [20]. The DEAP dataset is a benchmark dataset for emotion estimation research using EEG and has been widely used in many previous studies. Therefore, we also adopted this dataset for our research. It includes 32-channel EEG signals and 8-channel peripheral physiological signals recorded from 32 subjects as they watched 40 music videos, each with a duration of 63 s. The peripheral biosignals provide valuable insights into the participants’ physiological responses to the stimuli and include Galvanic Skin Response (GSR), heart rate (HR), and Electromyography (EMG). These signals measure changes in skin conductance, heart rate, and muscle activity, respectively, which are often associated with emotional arousal, stress, and emotional expression.

**Figure 2:**
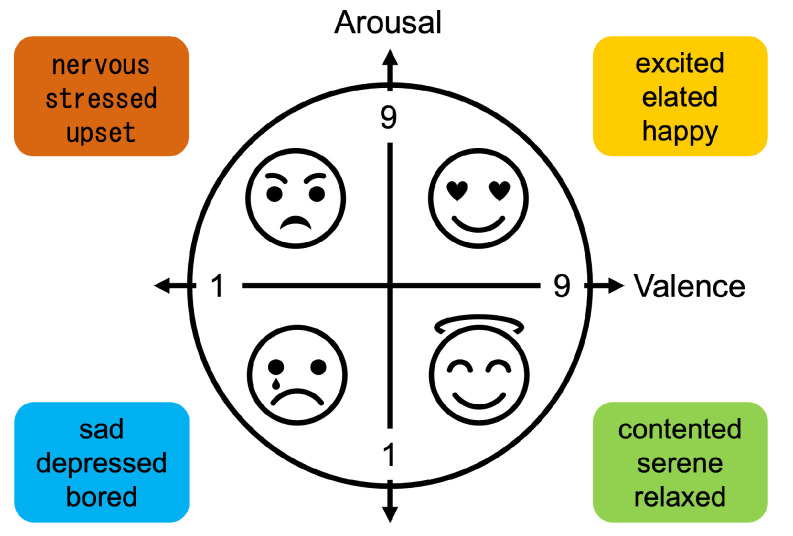
Russell’s circumplex model.

The music videos were selected in a pre-experiment to avoid bias in the elicited emotions. For each video, the EEG data consisted of 3 s baseline data and 60 s experimental data. The EEG data were measured with 32 electrodes installed in accordance with international regulations. After viewing the music videos, the 32 subjects completed subjective ratings of four evaluation items: arousal, valence, liking, and dominance. These ratings were obtained using a 9-point Likert scale, where 1 represents the lowest level, and 9 represents the highest. Arousal expresses the intensity of emotion, and valence indicates the degree of pleasantness or unpleasantness, both ranging from 1 to 9. Liking represents the degree of preference, while dominance is an index expressing the degree of emotional control or influence. Figure 2 illustrates Russell’s circumplex model of affect, a widely used model in emotion research. It provides a continuous and quantitative way to describe and measure emotional states. Russell’s model represents emotions on a two-dimensional plane, with valence (ranging from unpleasant to pleasant) as the horizontal axis and arousal (ranging from calm to excited) as the vertical axis. Russell’s model offers a comprehensive framework for describing and measuring emotional states in a continuous space. Table 1 shows the data arrays for each participant provided by DEAP. The sampling frequency was 512 Hz and was downsampled to 128 Hz. EOG artifacts were removed. Additionally, a bandpass filter was applied to provide data in the frequency range of 4–45 Hz.

In this paper, following previous studies, we do not use biological signals such as GSR, HR and EMG but only EEG data measured from all 32 electrodes provided by DEAP. For labels, of the four labels (arousal, valence, liking and dominance), only valence and arousal are used.

### 4.2. SEED Dataset

The SEED dataset, developed by the BCMI Laboratory at Shanghai Jiaotong University, is another widely used benchmark dataset for emotion recognition research using EEG signals [21,22]. This dataset was designed to investigate three distinct emotional states: neutral, negative, and positive.

The dataset consists of EEG recordings from 15 subjects participating in three separate experimental sessions on different days. This repeated measurement design enhances the reliability and generalizability of the collected data. Participants watched 15 carefully selected video clips to elicit specific emotional responses during each session. The experimental protocol for each video clip consisted of multiple segments: a 5 s movie prompt, followed by the main 4 min video content, a 45 s self-assessment period, and a 15 s rest interval. Each video clip was assigned an emotional label based on its content: −1 for negative emotions, 0 for neutral states, and 1 for positive emotions.

The EEG signals were initially recorded using 62 channels positioned according to the international 10–20 system and downsampled to 200 Hz. In our research, to ensure consistency with the DEAP dataset, we selected 32 electrodes from the original 62 channels corresponding to the electrode positions used in DEAP. Due to varying video durations, we extracted the middle 60 s from each approximately 4 min video recording for standardized analysis [28]. Table 2 presents the detailed structure of the SEED dataset after our electrode selection and temporal segmentation.

### 4.3. Feature Extraction of EEG Data

The preprocessing method for EEG data in this study follows the work of Yilong et al. and focuses on extracting time-domain features [39]. The EEG data for each video consist of 3 s of baseline EEG data recorded before watching the video and 60 s of EEG data recorded during the video viewing (the SEED dataset does not provide pre-stimulus EEG recordings; therefore, we employ the first 3 s of EEG data collected during the video presentation as the baseline condition). In this study, the EEG data before watching the video are defined as representing the basic emotional state. The preprocessing steps for the EEG data are as follows:

**Step 1**  Segmentation of EEG Data into 3 s Intervals
  The EEG data for each video are segmented into 3 s intervals. Thus, the EEG data are divided into 21 segments, with the first segment representing the baseline signal and the remaining 20 segments representing the EEG data during the video viewing.
**Step 2**  Calculation of Amplitude Changes between EEG Data and Baseline Signal
  The amplitude changes are calculated by subtracting the baseline signal from the EEG data during the video viewing. Finally, the data are standardized. In this study, these amplitude changes are used as time-domain features for the learning process.

### 4.4. Attention-Based PSO-LSTM Model

**Figure 3:**
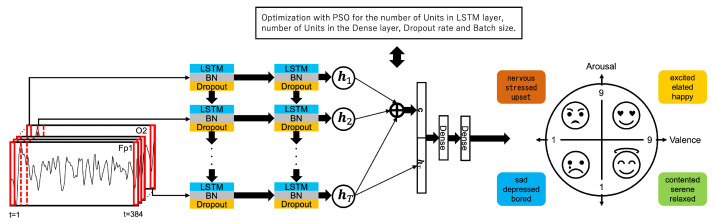
Attention-based PSO-LSTM.

In this study, we construct an emotion estimation model using LSTM to learn the temporal changes in EEG data, performing a four-class classification. The proposed emotion estimation model is illustrated in Figure 3. The LSTM layers are stacked in two layers, each using the tanh activation function. Additionally, Batch Normalization and dropout layers are inserted immediately after each LSTM layer to prevent overfitting.

The attention mechanism assigns weights to the hidden states of the LSTM for each time series. Subsequently, the context vector generated by the attention mechanism is concatenated with the final hidden state of the LSTM and then input into a fully connected layer with the tanh activation function. Finally, the four-class classification uses a fully connected layer with the softmax activation function.

PSO is employed to optimize the following five-dimensional parameters: the number of units in the first LSTM layer, the number of units in the second LSTM layer, the number of units in the dense layer, the dropout rate, and the batch size. Each parameter is searched within a predefined range, and the PSO algorithm identifies the optimal values. In this experiment, the predefined ranges for the hyperparameters are as follows: the number of units in the first LSTM layer (1–200), the number of units in the second LSTM layer (1–200), the number of units in the dense layer (1–200), the dropout rate (0.1–0.9), and the batch size (1–128).

In addition, the initial particle swarm is randomly initialized, and each particle’s parameter settings are used to train the model. Each particle evaluates its position based on the loss value, updating both its best position and the global best position of the swarm. Through iterations, the optimal parameter settings are expected to converge. The parameter settings that result in the minimum loss value are adopted, aiming to enhance the model’s performance. In this experiment, the categorical cross-entropy loss function is used to evaluate the performance of the model during training.

## 5. Experiment and Results

### 5.1. Overview

First, to validate the usefulness of the proposed model, its performance is evaluated through four-class emotion classification. Valence and arousal are represented by values ranging from 1 to 9; therefore, using 5 as a threshold, we perform four-class classification into High Valence–High Arousal (HVHA), Low Valence–High Arousal (LVHA), Low Valence–Low Arousal (LVLA), and High Valence–Low Arousal (HVLA). The performance of the proposed model is measured by the average accuracy across all subjects.

Next, to verify the usefulness of the attention mechanism, we compare the classification accuracy of the proposed model with that of the model without the Attention mechanism.

Finally, to verify the usefulness of PSO, the loss values for each iteration are plotted graphically to see if the loss values are decreasing. We also compare the classification accuracy of the model with fixed hyperparameters to see if it is optimized by PSO. Regarding the hyperparameters of the model, we set the number of LSTM units in the first layer to 128, the number of LSTM units in the second layer to 64, the number of units in the dense layer to 16, the dropout rate to 0.2, and the batch size to 32 [40].

### 5.2. Experiment Settings

The iteration number of the PSO is set to 10, and the number of particles is set to 15. The PSO weight parameters, *w*, $c_{1}$, and $c_{2}$, are set to 0.2, 0.3, and 0.5, respectively. With regard to the experimental conditions of the model, the number of epochs is set to 150. For the loss function, we use categorical cross-entropy. The optimization algorithm is Adam, with a learning rate of 0.0001. The evaluation metric is accuracy. To prevent overfitting caused by correlations in adjacent temporal segments of the input data, the input data are shuffled and then divided into 60% for training, 20% for validation, and 20% for testing. The classification accuracy of the proposed model is the average accuracy of the 32 subjects.

### 5.3. Experiment Results

Table 3 presents the classification accuracy of the proposed model. The model achieves an accuracy of 0.9404 in the four-class classification task using the DEAP dataset. This value surpasses the classification accuracies reported in previous studies, demonstrating the highest accuracy. Additionally, when using the SEED dataset, the model achieves an accuracy of 0.9732 in the three-class classification task.

Table 4 shows the classification accuracies of the model without the attention mechanism and the model with fixed hyperparameters. The model without the attention mechanism achieves an accuracy of 0.8382 for the DEAP dataset and 0.8814 for the SEED dataset. These values are approximately 0.1 lower than the proposed model for each dataset. Furthermore, the model with fixed hyperparameters achieves an accuracy of 0.9153 for the DEAP dataset and 0.9641 for the SEED dataset. These values are approximately 0.01–0.02 lower than the proposed model.

**Figure 4:**
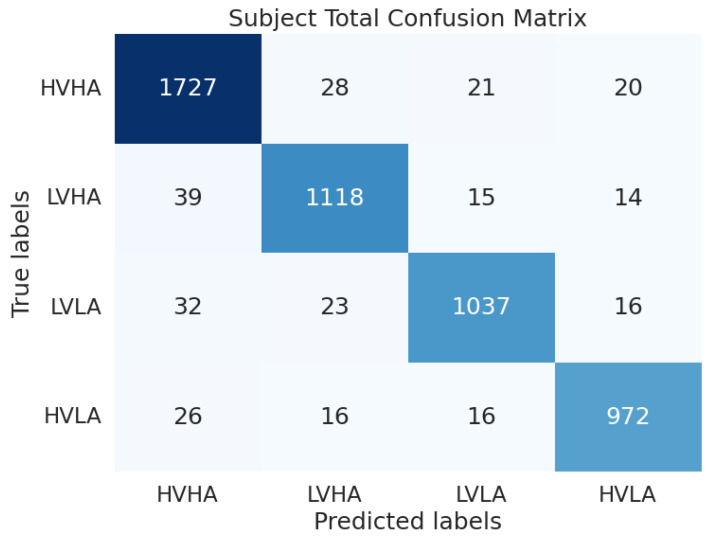
Confusion matrix of all 32 subjects on the DEAP dataset (The color intensity in the figure corresponds to the magnitude of the values, with larger numbers represented by darker shades of blue).

Figure 4 presents the element-wise aggregated confusion matrices across all subjects for the DEAP dataset when using the proposed model. The DEAP dataset has an imbalance, with more HVHA data. However, despite this data imbalance, the proposed model shows no significant bias, suggesting that it successfully extracts features and enables high-accuracy classification.

**Figure 5:**
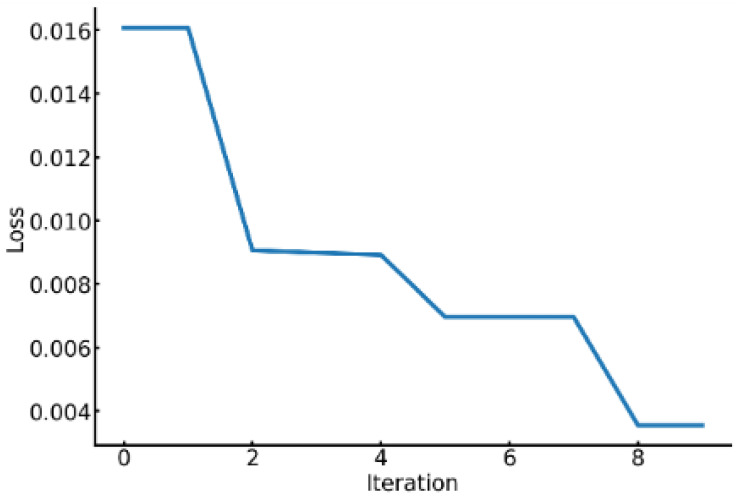
Loss curve of the PSO for Subject 1.

**Figure 6:**
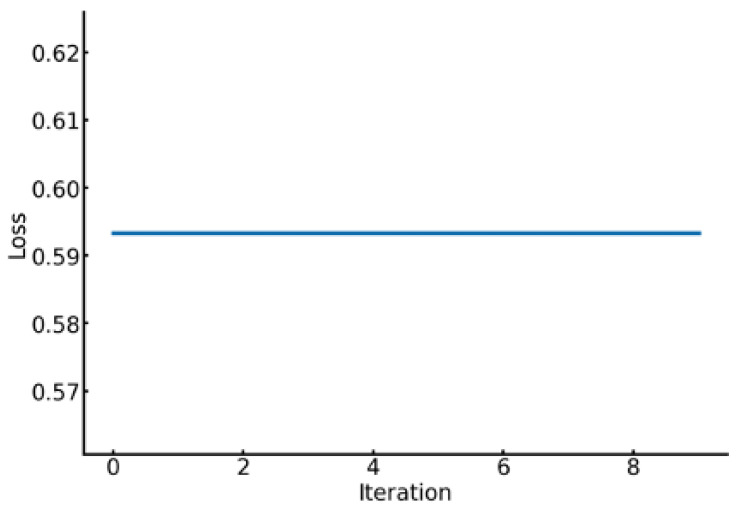
Loss curve of the PSO for Subject 17.

Figure 5 illustrates the progression of loss values per PSO iteration for Subject 1, who achieved a four-class classification accuracy of 0.9937 using the DEAP dataset. The graph clearly shows a steady decrease in loss values as iterations progress, indicating effective hyperparameter optimization by PSO. A sharp decrease in loss is observed during the initial iterations, followed by a consistent downward trend. Conversely, as shown in Figure 6, Subject 17’s loss values for the DEAP dataset remained constant per iteration, indicating stagnation in optimization. Subject 17’s four-class classification accuracy was 0.7624.

**Figure 7:**
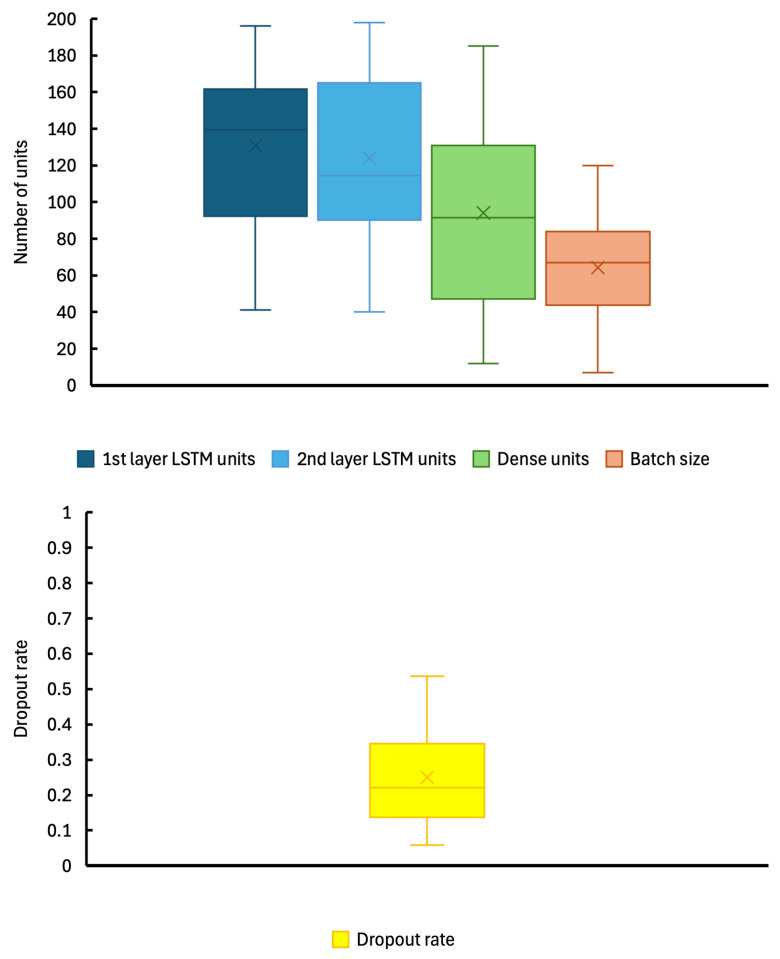
Box-and-whisker diagram for hyperparameters of all 32 subjects on the DEAP dataset.

Figure 7 shows box plots for each hyperparameter across all subjects in the DEAP datasets, highlighting significant inter-subject variability. Similar findings have been reported in prior studies, such as that of Bazargani et al. (2023), which demonstrated that subject-specific parameter tuning significantly improves model performance. The observed variation in hyperparameter values indicates that EEG data differences across individuals require tailored configurations for optimal results. These findings underscore the effectiveness of the PSO approach in identifying subject-specific optimal parameters and further highlight the necessity of personalized optimization in EEG-based emotion recognition.

### 5.4. Discussion

To the best of our knowledge, the state-of-the-art accuracy for four-class classification is 0.9100 as reported by Lin et al. [27]. The classification accuracy of 0.9404 achieved by the proposed model in four-class classification surpasses the performance reported in previous studies, strongly indicating the effectiveness of our approach. Furthermore, the model recorded a classification accuracy of 0.9732 in three-class classification using the SEED dataset, demonstrating the versatility of the proposed method.

Comparison with the model lacking the attention mechanism reveals that the attention mechanism contributes to an accuracy improvement of approximately 0.1. This difference suggests that the attention mechanism effectively emphasizes important features in EEG data, enabling the model to focus on more relevant information. Given that EEG data tend to contain noise and irrelevant information, the introduction of the attention mechanism likely reduced these influences, improving both the learning efficiency and model accuracy.

Comparison with the fixed-hyperparameter model confirms that PSO effectively optimizes the model’s hyperparameters, resulting in subject-specific models. For instance, in the case of Subject 1, PSO demonstrates its ability to converge toward the optimal solution by balancing exploration (searching broadly across different possibilities) and exploitation (focusing on the most promising options).

However, cases of stagnant optimization were observed, as exemplified by Subject 17. Possible causes include insufficient parameter search range and particle number in the current PSO experimental settings or a highly complex objective function making it difficult to escape local optima. Addressing these issues may require increasing the number of iterations and particles, expanding the search range, and implementing strategic initial particle placement.

Ten PSO iterations were chosen based on a balance between computational efficiency and empirical observations during preliminary experiments. While significant changes in the loss function for Subject 1 in the eighth iteration suggest ongoing optimization, the overall performance metrics indicate diminishing returns in improvement beyond 10 iterations for most subjects. Extending the iteration count further could potentially improve convergence for specific cases, such as Subject 1. However, it would increase the computational overhead. Future work could explore subject-specific iteration tuning or adaptive stopping criteria to address such scenarios more effectively.

The box plots of hyperparameters reveal that the optimal hyperparameter values differ significantly among subjects. This variability aligns with prior studies using EEG data, such as those by Lin et al. (2023) and Marjit et al. (2021), which reported similar inter-subject differences in classification performance and hyperparameter tuning. These differences reflect the unique individual EEG patterns and responses to stimuli, further emphasizing the importance of subject-specific model construction in improving accuracy.

While our proposed model achieved high classification accuracy overall, there remain areas for future improvement. These include enhancing the data preprocessing techniques, exploring alternative model architectures, and investigating more advanced optimization methods. A more detailed discussion of these future directions and the current limitations is provided in Section 6.

## 6. Limitations and Future Research

While the proposed attention-based PSO-LSTM model achieves strong results in emotion estimation from EEG data, several limitations need to be addressed. A possible limitation is that the PSO iteration count was fixed at 10 for all subjects without dynamic adjustment to individual optimization needs. Future work could investigate adaptive iteration tuning or stopping criteria to accommodate subject-specific variations better while balancing computational efficiency.

Another limitation involves the optimization stagnation observed in some cases, such as Subject 17. This could be attributed to insufficient iterations, an overly restricted search range, or the complexity of the objective function. Addressing these issues may involve increasing the number of iterations and particles, expanding the search range, or incorporating hybrid optimization techniques, such as combining PSO with evolutionary algorithms or simulated annealing.

Although EEG data preprocessing was carefully handled, EEG signals are inherently noisy. Exploring advanced noise reduction techniques, such as Independent Component Analysis (ICA), and implementing data augmentation could further enhance robustness. Data augmentation, a technique that artificially increases the diversity of training data by applying transformations such as scaling, cropping, or adding noise, could help the model generalize better to unseen data. Moreover, exploring alternative model architectures like transformer-based models, which have shown promise in time-series data, could enhance performance. Ensemble methods combining multiple models might also yield a more generalizable system.

The current study focuses on offline classification, which is not ideal for real-time applications. Future research could focus on reducing model latency and optimizing the system for real-time performance, potentially using model pruning techniques. Additionally, optimizing the model for edge computing would enable real-time deployment in devices with limited computational resources, which is essential for applications in healthcare or autonomous systems.

Finally, the model demonstrates significant subject-specific variability, highlighting the need for further personalization techniques. Transfer learning could allow the model to adapt quickly to new subjects without retraining, and on-the-fly model calibration methods could improve accuracy for new users. Addressing these limitations and expanding the model’s application to real-world contexts will further strengthen its performance and applicability.

## 7. Conclusions

This study introduces a novel attention-based PSO-LSTM model for emotion estimation from EEG data, achieving state-of-the-art accuracy in four-class emotion classification. The integration of attention mechanisms enhances the model’s ability to capture relevant temporal features, while PSO enables dynamic hyperparameter optimization. This combination successfully addresses key challenges in EEG-based emotion recognition, particularly in managing noise and temporal pattern complexity.

Our user-dependent approach effectively constructs subject-specific models that capture individual variations in EEG patterns, demonstrating significant advancements over fixed-hyperparameter approaches. However, this comes at the cost of broader generalizability, reflecting a fundamental trade-off in EEG-based emotion recognition: balancing individual accuracy with widespread applicability.

The model’s success in adapting to individual differences while maintaining robust performance suggests promising applications in healthcare, neuromarketing, and human–computer interaction, where accurate emotion recognition is essential. Moving forward, the development of user-independent models that maintain high individual accuracy while functioning effectively across subjects represents the next frontier in this field.

This study lays the groundwork for more versatile emotion recognition systems that can bridge the gap between individual optimization and broad applicability, advancing our understanding of human emotion and its practical applications in real-world scenarios.

## Figures and Tables

**Table 1:** DEAP arrays of each participant.

Array Name	Array Shape	Array Contents
data	40 × 32 × 8064	video/trial × channel × data
labels	40 × 4	video/trial × label (valence, arousal, liking, dominance)

**Table 2:** SEED arrays of each participant.

Array Name	Array Shape	Array Contents
data	45 × 32 × 12,000	video/trial × channel × data
labels	45 × 3	video/trial × label (negative, neutral, positive)

**Table 3:** Experimental results and comparison with previous research.

Research	Model	DEAP	SEED
Deng X et al. [28]	SFE-Net	0.7423	0.9919
Marjit et al. [23]	MLP	0.8352	-
Lin et al. [27]	CSGNN	0.9100	0.9022
Proposed model	Attention-based PSO-LSTM	0.9404	0.9732

**Table 4:** Classification results using the proposed model.

Dataset	Method	Mean	Std	Max	Min
DEAP	Without attention	0.8382	0.0711	0.9499	0.6687
	Fixed parameters	0.9153	0.0437	0.9812	0.7875
	Proposed model	0.9404	0.0492	0.9937	0.7624
SEED	Without attention	0.8814	0.0638	0.9611	0.7222
	Fixed parameters	0.9641	0.0288	1.000	0.8999
	Proposed model	0.9732	0.0261	1.000	0.9166

## Data Availability

The DEAP dataset can be found at https://www.eecs.qmul.ac.uk/mmv/datasets/deap/ (accessed on 7 November 2024).

## References

[1] Dzedzickis A., Kaklauskas A., Bucinskas V. (2020). Human Emotion Recognition: Review of Sensors and Methods. Sensors.

[2] Quiles M., Martínez Beltrán E., López Bernal S., Prat E., Campo L., Fernández-Maimó L., Huertas A. (2024). Data fusion in neuromarketing: Multimodal analysis of biosignals, lifecycle stages, current advances, datasets, trends, and challenges. Inf. Fusion.

[3] Abuín Vences N., Díaz-Campo J., García Rosales D.F. (2020). Neuromarketing as an Emotional Connection Tool Between Organizations and Audiences in Social Networks. A Theoretical Review. Front. Psychol..

[4] Li X., Song D., Zhang P., Zhang Y., Hou Y., Hu B. (2018). Exploring EEG features in cross-subject emotion recognition. Front. Neurosci..

[5] Katsis C., Katertsidis N., Fotiadis D. (2011). An integrated system based on physiological signals for the assessment of affective states in patients with anxiety disorders. Biomed. Signal Process. Control.

[6] Guo R., Guo H., Wang L., Chen M., Yang D., Li B. (2024). Development and application of emotion recognition technology—A systematic literature review. BMC Psychol..

[7] Hossain M.S., Muhammad G. (2019). Emotion recognition using deep learning approach from audio–visual emotional big data. Inf. Fusion.

[8] Venkatesh Y., Kassim A., Nguyen T.D. (2012). On the simultaneous recognition of identity and expression from BU-3DFE datasets. Pattern Recognit. Lett..

[9] Samal P., Hashmi M. (2024). Role of machine learning and deep learning techniques in EEG-based BCI emotion recognition system: A review. Artif. Intell. Rev..

[10] Hamzah H.A., Abdalla K.K. (2024). EEG-based emotion recognition systems; comprehensive study. Heliyon.

[11] Kumar G.S., Shashi A.A., Sampathila N., Vinoth R. (2022). Machine Learning Models for Classification of Human Emotions Using Multivariate Brain Signals. Computers.

[12] Wang X., Ren Y., Luo Z., He W., Hong J., Huang Y. (2023). Deep learning-based EEG emotion recognition: Current trends and future perspectives. Front. Psychol..

[13] Alhagry S., Aly A., El-Khoribi R. (2017). Emotion Recognition based on EEG using LSTM Recurrent Neural Network. Int. J. Adv. Comput. Sci. Appl..

[14] Çimtay Y., Ekmekcioglu E. (2020). Investigating the Use of Pretrained Convolutional Neural Network on Cross-Subject and Cross-Dataset EEG Emotion Recognition. Sensors.

[15] Ayata D., Yaslan Y., Kamasak M. Emotion Recognition via Multi Channel EEG Signal Fusion and Pattern Recognition. Proceedings of the 25th European Signal Processing Conference Multimodal Processing, Modeling and Learning Approaches for Human-Computer/Robot Interaction (EUSIPCO).

[16] Su Y., Liu Y., Xiao Y., Ma J., Li D. (2024). A review of artificial intelligence methods enabled music-evoked EEG emotion recognition and their applications. Front. Neurosci..

[17] Hochreiter S., Schmidhuber J. (1997). Long Short-Term Memory. Neural Comput..

[18] Lin S.W., Ying K.C., Chen S.C., Lee Z.J. (2008). Particle swarm optimization for parameter determination and feature selection of support vector machines. Expert Syst. Appl..

[19] Vempati R., Sharma L.D. (2023). A systematic review on automated human emotion recognition using electroencephalogram signals and artificial intelligence. Results Eng..

[20] Koelstra S., Muehl C., Soleymani M., Lee J.S., Yazdani A., Ebrahimi T., Pun T., Nijholt A., Patras I. (2012). DEAP: A Database for Emotion Analysis using Physiological Signals. IEEE Trans. Affect. Comput..

[21] Zheng W.L., Lu B.L. (2015). Investigating Critical Frequency Bands and Channels for EEG-based Emotion Recognition with Deep Neural Networks. IEEE Trans. Auton. Ment. Dev..

[22] Duan R.N., Zhu J.Y., Lu B.L. Differential entropy feature for EEG-based emotion classification. Proceedings of the 2013 6th International IEEE/EMBS Conference on Neural Engineering (NER).

[23] Marjit S., Talukdar U., Hazarika S.M. EEG-Based Emotion Recognition Using Genetic Algorithm Optimized Multi-Layer Perceptron. Proceedings of the 2021 International Symposium of Asian Control Association on Intelligent Robotics and Industrial Automation (IRIA).

[24] Ajith K., Menaka R., Kumar S.S. (2021). EEG based mental state analysis. J. Phys. Conf. Ser..

[25] Nandi A., Xhafa F., Subirats L., Fort S. (2021). Real-Time Emotion Classification Using EEG Data Stream in E-Learning Contexts. Sensors.

[26] Bazargani M., Tahmasbi A., Yazdchi M., Baharlouei Z. (2023). An Emotion Recognition Embedded System using a Lightweight Deep Learning Model. J. Med. Signals Sens..

[27] Lin X., Chen J., Ma W., Tang W., Wang Y. (2023). EEG emotion recognition using improved graph neural network with channel selection. Comput. Methods Programs Biomed..

[28] Deng X., Zhu J., Yang S. SFE-Net: EEG-based Emotion Recognition with Symmetrical Spatial Feature Extraction. Proceedings of the MM ’21: Proceedings of the 29th ACM International Conference on Multimedia.

[29] Kan H., Yu J., Huang J., Liu Z., Wang H., Zhou H. (2023). Self-supervised Group Meiosis Contrastive Learning for EEG-based Emotion Recognition. Appl. Intell..

[30] Garg A., Kapoor A., Bedi A., Sunkaria R. Merged LSTM Model for emotion classification using EEG signals. Proceedings of the 2019 4th International Conference on Information Systems and Computer Networks (ISCON).

[31] Li Y., Huang J., Zhou H., Zhong N. (2017). Human Emotion Recognition with Electroencephalographic Multidimensional Features by Hybrid Deep Neural Networks. Appl. Sci..

[32] Lipton Z.C., Berkowitz J., Elkan C. (2015). A critical review of recurrent neural networks for sequence learning. arXiv.

[33] Greff K., Srivastava R., Koutník J., Steunebrink B., Schmidhuber J. (2015). LSTM: A search space odyssey. IEEE Trans. Neural Netw. Learn. Syst..

[34] Poli R., Kennedy J., Blackwell T. (2007). Particle swarm optimization. Swarm Intell..

[35] Gad A. (2022). Particle Swarm Optimization Algorithm and Its Applications: A Systematic Review. Arch. Comput. Methods Eng..

[36] Kennedy J., Eberhart R. Particle swarm optimization. Proceedings of the ICNN’95—International Conference on Neural Networks.

[37] Vaswani A., Shazeer N., Parmar N., Uszkoreit J., Jones L., Gomez A., Kaiser L., Polosukhin I. (2017). Attention Is All You Need. arXiv.

[38] Zhang G., Davoodnia V., Sepas-Moghaddam A., Zhang Y., Etemad S.A. (2019). Classification of Hand Movements from EEG using a Deep Attention-based LSTM Network. IEEE Sens. J..

[39] Yang Y., Wu Q., Qiu M., Wang Y., Chen X. Emotion Recognition from Multi-Channel EEG through Parallel Convolutional Recurrent Neural Network. Proceedings of the 2018 International Joint Conference on Neural Networks (IJCNN).

[40] Kim Y., Choi A. (2020). EEG-Based Emotion Classification Using Long Short-Term Memory Network with Attention Mechanism. Sensors.

